# Synthesis of a CCNC–Silica–Graphene Oxide Porous Monolith for Efficient Copper Ion Removal

**DOI:** 10.3390/gels11100832

**Published:** 2025-10-17

**Authors:** Nduduzo Khumalo, Samson Mohomane, Vetrimurugan Elumalai, Tshwafo Motaung

**Affiliations:** 1 Department of Chemistry, University of Zululand, KwaDlangezwa Campus, Private Bag X1001, KwaDlangezwa 3886, South Africa; khumalonl@unizulu.ac.za; 2 Department of Hydrology, University of Zululand, KwaDlangezwa Campus, Private Bag X1001, KwaDlangezwa 3886, South Africa; 3 Department of Chemistry and Chemical Technology, Sefako Makgatho Health Science University, P.O. Box 94, Medunsa 0204, South Africa

**Keywords:** cellulose nanocrystals, porous monolith, adsorption, wastewater treatment, graphene oxide, hybrid composite

## Abstract

Heavy metal contamination in water, predominantly from copper (Cu(II)) ions, poses substantial risks to human and environmental health. This study developed a novel, robust adsorbent known as a carboxylate cellulose nanocrystal–silica–graphene oxide hybrid composite porous monolith, which effectively removes Cu(II) from water in a rapid manner. Carboxylate cellulose nanocrystals with enhanced metal-binding properties were synthesized from cellulose extracted from sugarcane bagasse, a significant agricultural byproduct. The porous monolith was synthesized through the combination of carboxylate cellulose nanocrystals, tetraethyl orthosilicate (TEOS), and graphene oxide, utilizing a sol–gel method. The efficacy of the synthesis was confirmed using Fourier-Transform Infra-red (FTIR), X-ray diffraction (XRD), thermogravimetric analysis (TGA), scanning electron microscope (SEM), and Brunauer–Emmett–Teller (BET) analyses. The material exhibited a highly porous mesoporous structure with a surface area of 512 m^2^/g, signifying a significant enhancement. Batch adsorption experiments under optimal conditions (pH = 5.5, contact time = 240 min, initial concentration = 200 mg/L) demonstrated a high experimental adsorption capacity of 172 mg/g for Cu(II). The adsorption process was best described by the Langmuir isotherm model, which yielded a theoretical maximum capacity (q_m_) of 172 mg/g, and the pseudo-second-order kinetic model, confirming monolayer coverage and chemisorption as the rate-limiting step. Thermodynamic analyses demonstrate that the process is both spontaneous and exothermic. The porous monolith demonstrates the capability for multiple uses, maintaining over 70% efficiency after five cycles. The findings indicate that the carboxylate cellulose nanocrystal–silica–graphene oxide hybrid composite porous monolith is an efficient and robust method for the remediation of copper-contaminated water.

## 1. Introduction

Heavy metal contamination of aquatic ecosystems poses a significant threat to global ecology. Copper (Cu(II)) is a significant contaminant due to its extensive application in electronics production, electroplating, plumbing, and as a component in insecticides and fungicides for agriculture [1,2]. Human activities are the primary contributors of copper contamination. They emit waste that can infiltrate soil and water systems via runoff, industrial discharge, and inadequate waste management. Heavy metals such as lead (Pb), cadmium (Cd), and mercury (Hg) might be more detrimental at lower concentrations. Copper, conversely, is among the most prevalent metals included in industrial and agricultural waste due to its frequent utilization. For instance, in numerous streams of mining and technological waste, the concentration of copper can exceed that of more hazardous elements such as cadmium by one to two orders of magnitude. This renders it a high-volume pollutant that requires prompt removal. Copper is essential in minimal quantities; nevertheless, excessive levels in water and soil are detrimental to ecosystems and human health. Prolonged exposure to humans can cause significant harm to the liver and kidneys. In aquatic ecosystems, it can be detrimental, impairing cellular functions and leading to population decreases [2,3]. It is essential to identify effective methods for removing copper from contaminated water to mitigate its environmental impact and associated health risks. Therefore, it is crucial to find effective methods to remove copper from polluted water.

Heavy metal contamination, responsible for numerous diseases and disorders, has emerged as a significant environmental issue [1,2]. Copper(II) ions are predominantly located in wastewater and contaminated soil [2,3]. The improper disposal of Cu(II) ions has resulted in significant environmental contamination, complicating the utilization of surface water and groundwater [4]. Adsorption [5] chemical precipitation [6], reverse osmosis [7], and ion exchange [8]. There are prevalent methods for the removal of metal ions. Adsorption is an effective method for the removal of heavy metal ions from wastewater due to its simplicity, cost-effectiveness, and efficiency [4,8]. Adsorbent materials are essential for the adsorption process, and the application of natural materials in water treatment has attracted considerable attention. A growing quantity of adsorbents is being manufactured from cost-effective natural resources [9].

Recent studies have examined several nanomaterials for water purification due to their large surface area, unique physicochemical properties, and improved adsorption efficacy [10]. Cellulose nanocrystals (CNCs) have copper (Cu(II)) ion adsorption capacities ranging from 20 to 100 mg/g, whereas functionalized carboxylate CNCs often exceed 100 mg/g due to enhanced binding sites [10]. CNCs are derived from renewable biomass, are biodegradable, and possess excellent mechanical qualities, rendering them suitable for environmental applications [11]. The incorporation of carboxyl groups onto CNCs enhances their surface charge and active binding sites, significantly augmenting their capacity to adsorb heavy metals [12,13].

Silica-based materials have demonstrated potential for the adsorption of heavy metals. Mesoporous silica materials, such as MCM-41 and SBA-15, demonstrate Cu(II) adsorption capabilities between 10 and 60 mg/g [14]. The elevated surface area and modifiable pore architectures of these materials facilitate the adsorption process [15]. Standard silica gel generally adsorbs 10 to 30 mg/g of Cu(II), although its efficacy can be markedly enhanced via surface functionalization with chelating chemicals, which augment its affinity for metal ions [16,17]. The incorporation of cellulose and silica into composite materials enhances their adsorption capabilities due to the synergistic interaction between the two constituents. Composites composed of cellulose and silica can adsorb between 50 and 150 mg/g of adsorbate, contingent upon the cellulose-to-silica ratio and the surface functional groups [18,19]. These composites leverage the strength and biodegradability of cellulose, along with the extensive surface area and stability of silica, to provide efficient and durable adsorbents for the removal of heavy metals.

Advanced hybrid nanocomposites demonstrate potential; however, many current adsorbents face obstacles, such as moderate adsorption capacities, insufficient structural integrity leading to difficult recovery and reduced reusability, and diminished effectiveness at near-neutral pH levels, common in various wastewaters. We propose that the synergistic integration of carboxylate cellulose nanocrystals (CCNCs), silica (SiO_2_), and graphene oxide (GO) within a three-dimensional porous monolith matrix will significantly enhance Cu(II) adsorption efficiency, stability, and reusability.

Multiple studies have investigated binary composites for the removal of heavy metals. Cellulose–silica composites have been reported for dye adsorption [20], whilst graphene oxide–cellulose materials have shown potential for metal ion absorption [21,22]. However, the integration of functionalized cellulose, silica, and graphene oxide into a cohesive, monolithic porous monolith specifically designed for enhanced Cu(II) adsorption remains mainly unexplored. Numerous contemporary adsorbents exist as either powders or weak gels, complicating their retrieval and subsequent reuse in continuously operating systems. Furthermore, while the individual components are acknowledged, the synergistic effects arising from their tripartite integration within a 3D porous network, particularly the interaction of the carboxylate groups of CCNCs, the structural stability of silica, and the surface functionality of GO to enhance capacity, kinetics, and reusability are insufficiently investigated and quantified.

This study addresses a specific gap by developing and characterizing a novel ternary CCNC-SiO_2_-GO hybrid porous monolith. We hypothesize that the synergistic amalgamation of these components will provide a resilient, monolithic adsorbent with superior Cu(II) adsorption capacity, kinetics, and reusability, exceeding the performance of its individual components or binary composites. This research is novel due to: (1) the innovative design of a ternary porous monolith derived from sustainable sugarcane bagasse; (2) a comprehensive analysis of the synergistic adsorption mechanism involving the high density of carboxylate groups (CCNC), the mesoporous scaffold (SiO_2_), and the enhanced surface area/functionality (GO); and (3) a detailed performance evaluation demonstrating high efficiency and remarkable regenerability over multiple cycles. This study presents innovative insights into the design of multifunctional, biomass-derived porous monoliths for efficient and practical wastewater remediation. We hypothesize that the synergistic integration of carboxylate cellulose nanocrystals (CCNCs), silica (SiO_2_), and graphene oxide (GO) into a three-dimensional porous monolith will result in a robust adsorbent with superior Cu(II) adsorption capacity, faster kinetics, and enhanced reusability compared to its individual components or binary composites, due to the combined effects of high carboxylate group density, mesoporous scaffolding, and enhanced surface functionality.

The main aim of this study was to synthesize, describe, and evaluate a novel carboxylate cellulose nanocrystal–silica–graphene oxide (CCNC-SiO_2_-GO) hybrid porous monolith for the efficient removal of Cu(II) ions from aqueous solutions. The specific objectives were to (1) extract cellulose from sugarcane bagasse (SCB) and synthesize cellulose-based nanocrystals (CCNCs) with improved metal-binding capacity; (2) create a monolithic CCNC-SiO_2_-GO porous monolith through a sol–gel process; (3) thoroughly characterize the chemical, structural, and thermal properties of the synthesized materials using FTIR, XRD, SEM, TGA, and BET analysis; (4) assess the Cu(II) adsorption performance by analyzing the influence of key parameters such as contact time, pH, initial concentration, and adsorbent dosage; and (5) evaluate the adsorption kinetics, isotherms, thermodynamics, and reusability of the porous monolith to clarify the underlying removal mechanism.

## 2. Results and Discussion

### 2.1. FTIR Spectroscopy Analysis

**Figure 1:**
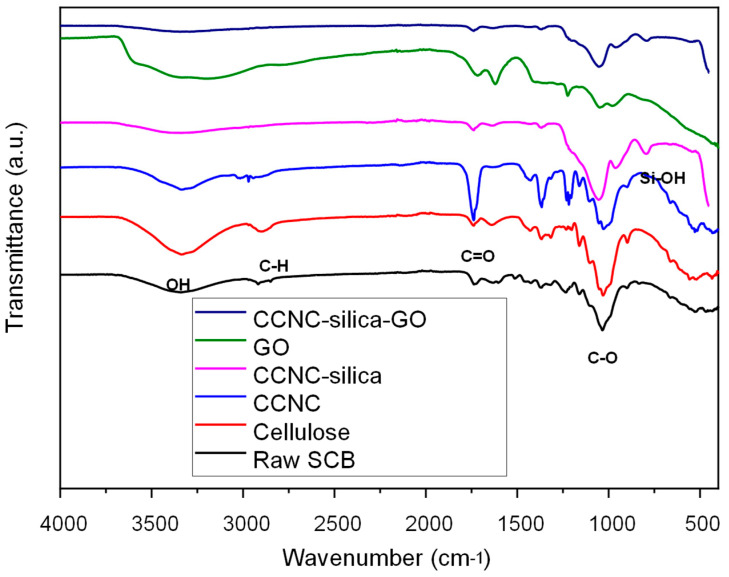
FTIR spectra of the synthesized materials: raw sugarcane bagasse (SCB), extracted cellulose, carboxylate cellulose nanocrystals (CCNC), CCNC–silica porous monolith, graphene oxide (GO), and CCNC–silica–GO hybrid composite porous monolith.

The synthesis of the carboxylate cellulose–silica graphene oxide (GO) hybrid nanocomposite was validated using FTIR analysis. The FTIR spectra presented in Figure 1 includes data for raw sugarcane bagasse (SCB), extracted cellulose, carboxylate cellulose nanocrystals (CCNC), CCNC–silica porous monolith, GO, and the CCNC–silica–GO porous monolith. In raw SCB, characteristic peaks indicative of natural fibers were observed at 3325 cm^−1^ (─OH stretching), 2896 cm^−1^ (C─H vibrations), 1361 cm^−1^ (─CH_2_ bending), 1025 cm^−1^ (C─O stretching), and 897 cm^−1^ (─CH_2_ bending), corresponding to cellulose I [23,24].

An additional peak at approximately 1312 cm^−1^ was distinctly present in extracted cellulose and CCNC but absent in raw SCB. This peak is likely due to C─H wagging, attributed to disrupted hydrogen bonds [20]. The extracted cellulose exhibited identical peak patterns to raw SCB, in addition to the peak at 1312 cm^−1^. It lacked peaks at 1508 and 1239 cm^−1^; however, it exhibited increased intensity at 897 cm^−1^. This indicates that the amorphous regions were eliminated and β-glycosidic connections were established between glucose units in cellulose [20,24].

The FTIR spectra of CCNC exhibited a significant increase in the peak intensity of the carbonyl group at 1740 cm^−1^ [25]. The FTIR spectra of the CCNC–silica porous monolith displayed a significant peak at 793 cm^−1^, indicative of the stretching and bending vibrations of the silanol group (Si-OH) [21]. Peaks were seen at 1361 cm^−1^, 1215 cm^−1^, and 1044 cm^−1^, corresponding to C–O alkoxy stretching, C–O–C asymmetric stretching, and C–O epoxy stretching, respectively. This demonstrated the successful fabrication of cellulose-based silica porous monoliths.

A broad O-H stretching peak (3200–3550 cm^−1^) indicated the presence of hydroxyl groups from both cellulose and graphene oxide (GO). The C=O stretching peak at approximately 1720 cm^−1^ indicated the presence of GO. The peaks near 1050 cm^−1^ were broadened due to the simultaneous occurrence of Si-O-Si stretching (from silica) and C-O stretching (from cellulose and graphene oxide). The presence of Si-OH and Si-O-Si stretching peaks confirmed the incorporation of silica into the nanocomposite [21].

### 2.2. X-Ray Diffraction Analysis

**Figure 2:**
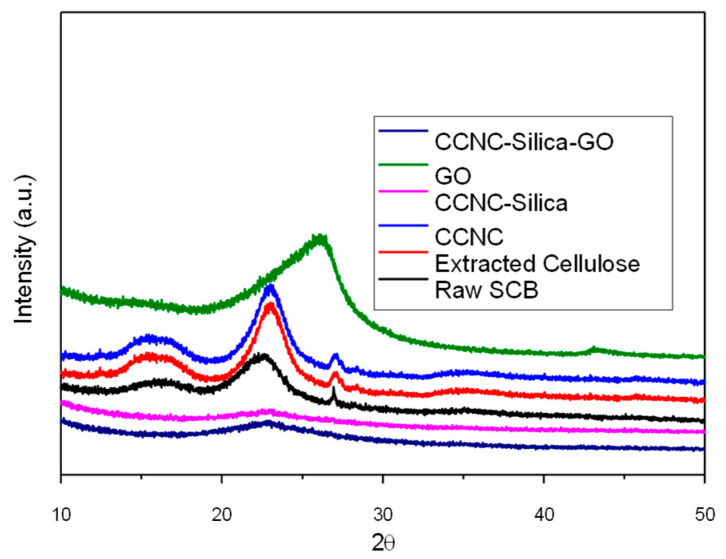
XRD spectra of raw SCB, extracted cellulose, carboxylated cellulose, carboxylated CNC–silica porous monolith, GO, and carboxylated CNC–silica–GO porous monolith hybridized composite material.

Figure 2 depicts the XRD analysis of raw sugarcane bagasse, extracted cellulose, carboxylate cellulose nanocrystals (CCNC), CCNC-SiO_2_ porous monolith, graphene oxide (GO), and CCNC-SiO_2_-GO porous monoliths. Every stage of material processing and synthesis displays unique structural attributes. The XRD pattern of unprocessed sugarcane bagasse exhibits prominent peaks at 2θ = 15° (110 plane) and 22° (200 plane). This indicates that it is semi-crystalline due to the presence of cellulose, hemicellulose, and lignin [22]. The XRD pattern displays more prominent peaks at 2θ = 22.5° (200 plane) after the elimination of cellulose. This indicates that the crystalline cellulose I structure remains intact, but amorphous hemicellulose and lignin have been eliminated [26]. A pronounced peak at 2θ = 22.5° (200 plane) for CCNC indicates that the nanocrystals exhibit enhanced order and a greater degree of crystallinity [25]. The CCNC-SiO_2_ porous monolith maintains the cellulose I peak at 2θ = 22.5° (200 plane) and displays new broad peaks at 2θ = 20° (amorphous SiO_2_) and 26° (amorphous SiO_2_). This indicates the successful integration of SiO_2_ [27]. It is important to distinguish the origin of the diffraction feature near 26° observed in different samples. In the raw SCB, the broad feature in this region is attributed to the overlapping (004) reflection of cellulose I and the complex, semi-crystalline structure of lignin [28,29]. In extracted cellulose and CCNC, this feature is solely due to the (004) plane of the cellulose I crystalline lattice [27]. In contrast, the sharp peak at ~26° in the graphene oxide (GO) sample corresponds to the (002) plane, representing the restacked graphitic domains with an interlayer spacing that is larger than pristine graphite due to the presence of oxygenated functional groups [30]. Finally, in the CCNC-SiO_2_ and CCNC-SiO_2_-GO porous monoliths, the broad feature centered around 26° is characteristic of the amorphous silica (SiO_2_) network, which produces a diffuse halo in this region [31]. Therefore, while a peak appears near 26° in multiple diffractograms, it arises from distinct crystalline (cellulose, GO) and amorphous (silica) components, reflecting the successful integration of all materials into the final composite. This indicates that a composite porous monolith with enhanced characteristics has been successfully produced [27]. This analysis demonstrates the progression of material structures throughout time and the successful modification and incorporation of diverse components to produce advanced composite materials.

The Crystallinity Index (CI) quantifies alterations in the XRD patterns. The process ascends from raw bagasse to extracted cellulose and descends with each alteration (Table 1). Carboxylate cellulose, formed through the introduction of carboxyl groups, has broader and less strong peaks, indicating partial amorphization [32]. The incorporation of amorphous silica further reduces the CI, as demonstrated in the porous monoliths. The peak placements remain largely unchanged; however, the heights and widths of the summits vary. This indicates that crystalline and structural disturbance vary at each processing stage. These findings highlight the structural alterations necessary for understanding the material properties and potential uses of modified cellulose derived from sugarcane bagasse [33].

### 2.3. SEM Analysis

**Figure 3:**
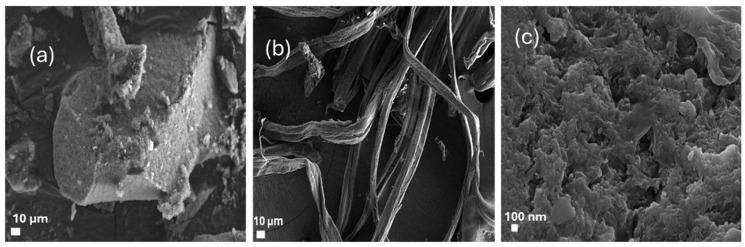
SEM images of (**a**) raw SCB, (**b**) extracted cellulose, (**c**) carboxylated CNC–silica–GO porous monolith hybridized composite material.

Figure 3 presents SEM images demonstrating substantial morphological variations that transpire when raw sugarcane bagasse (SCB) is transformed into the synthetic CCNC–silica–GO porous monolith. The unrefined SCB (Figure 3a) exhibits a coarse, thick, and fibrous surface, characteristic of lignin, hemicellulose, and amorphous constituents tightly integrated within the cellulose matrix [22,26]. The cellulose acquired post-chemical extraction (Figure 3b) exhibits a purer, fibrillated structure with less surface contaminants. This indicates that lignin and hemicellulose were effectively eliminated, exposing a better-organized cellulose microfibrillar network [20,24]. In contrast, the CCNC–silica–GO porous monolith (Figure 3c) demonstrates a highly porous, sponge-like morphology, marked by interconnected and uniformly distributed pores. The morphology results from the synergistic interaction of CCNCs, silica, and graphene oxide during sol–gel processing, which inhibits agglomeration and facilitates the development of an open porous structure [18,34]. The hierarchical porosity improves adsorption through an increased surface area and promotes the transport of metal ions into the porous monolith matrix [19,35]. The findings demonstrate that raw biomass can be converted into a functional hybrid porous monolith exhibiting improved textural properties appropriate for water purification.

### 2.4. TGA

**Figure 4:**
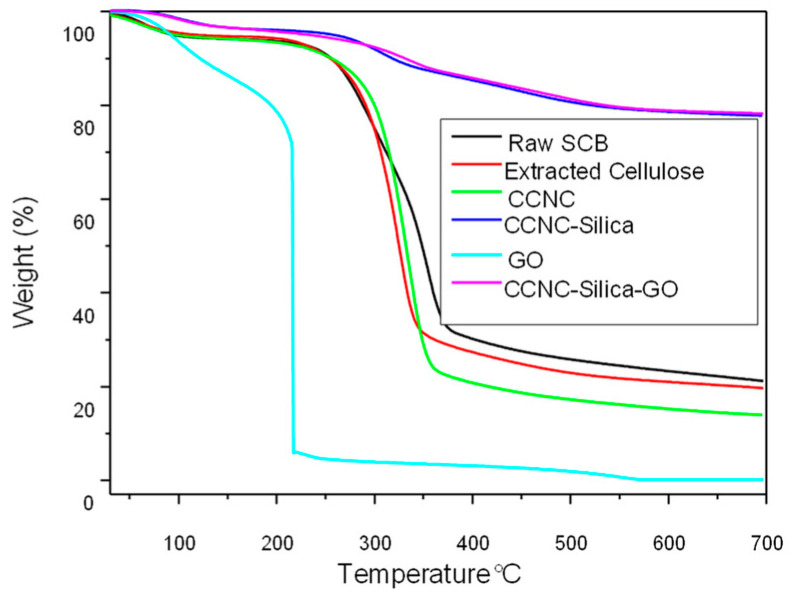
TGA spectra of raw SCB, extracted cellulose, carboxylated cellulose, carboxylated CNC–silica porous monolith, GO, and carboxylated CNC–silica–GO porous monolith hybridized composite material.

**Figure 5:**
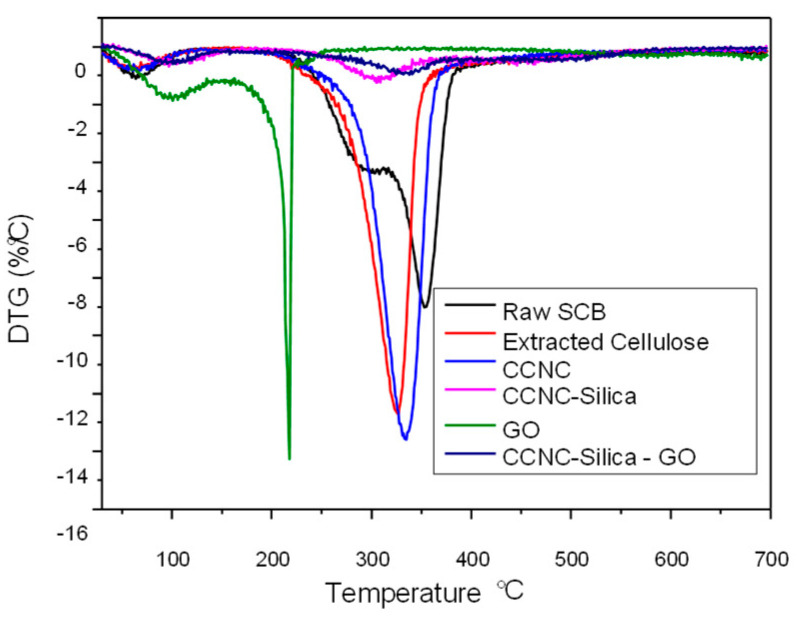
DTG spectra of raw SCB, extracted cellulose, carboxylated cellulose, carboxylated CNC–silica porous monolith, GO, and carboxylated CNC–silica–GO porous monolith hybridized composite material.

The TGA and DTG analyses furnish comprehensive insights into the thermal behavior and degradation characteristics of raw sugarcane bagasse, extracted cellulose, carboxylate cellulose nanocrystals (CCNC), CCNC-SiO_2_ porous monolith, graphene oxide (GO), and CCNC-SiO_2_-GO porous monoliths (refer to Figure 4 and Figure 5). The TGA plot for raw sugarcane bagasse indicates that it decomposes in three stages. Initially, moisture evaporates at approximately 100 °C. Subsequently, hemicellulose and some cellulose components decompose within the temperature range of 270 to 390 °C. Ultimately, cellulose and lignin decompose within the temperature range of 320 to 354 °C. The DTG peaks occur at around 300 °C and 354 °C [28,36]. Extracted cellulose exhibits superior thermal stability, demonstrating minimal initial weight loss and primary degradation occurring between 300 and 326 °C, as indicated by a DTG peak around 320–325 °C [29,31]. CCNC exhibits superior thermal stability, decomposing between 310 and 334 °C, with a DTG peak occurring between 335 and 340 °C. This results from carboxylate groups and the degradation of cellulose [10,30].

GO illustrates numerous methods by which systems might fail when temperatures increase. The use of SiO_2_ with CCNC enhances its thermal stability. The primary weight loss occurs between 400 and 453 °C, indicating the degradation of organic components while SiO_2_ remains constant [37]. For instance, it experiences significant weight loss between 150 and 250 °C due to the decomposition of its oxygen-containing functional groups. A DTG peak is observed between 200 and 250 °C [38,39]. The TGA and DTG measurements of the CCNC-SiO_2_-GO porous monolith reveal a distinct pattern of thermal degradation, indicating successful composite formation due to the synergistic interaction of its components. Porous monolith exhibits superior thermal stability compared to merely combining its components, with primary disintegration occurring between 300 and 400 °C. The silica matrix and graphene oxide sheets serve as physical barriers that prevent the escape of volatile breakdown products from the cellulose. Robust hydrogen bonding among the components establishes a cross-linked network that facilitates stable char formation. Graphene oxide accelerates char formation, hence safeguarding the substance. The broad DTG peak seen between 340 and 380 °C indicates simultaneous degradation of the CCNC and GO functional groups. The silica framework maintains structural stability, resulting in a robust composite that endures elevated temperatures [40,41].

The most significant distinction between the composites and their constituent components is the residual mass remaining after incineration at 700 °C. The organic components (CCNC and GO) predominantly decompose, resulting in minimal residual material (5–25%). Nonetheless, the CCNC-SiO_2_ and CCNC-SiO_2_-GO porous monoliths have a comparatively elevated residual mass of approximately 80%. This phenomenon cannot be elucidated by a mere additive action of the components. It is instead associated with a synergistic mechanism that generates char. The silica matrix and graphene oxide sheets function as both a physical barrier and a catalyst. They inhibit the release of volatile degradation products from cellulose and facilitate the conversion of organic carbon into a stable, carbonaceous char, which is encapsulated and reinforced by the thermally stable silica matrix. This process efficiently sequesters a substantial quantity of carbon that would otherwise be released. It transforms carbon from a combustible material into a stable component of inorganic waste. The significant increase in residual mass serves as compelling evidence that an integrated composite material with distinct properties from its constituent components has been effectively created.

### 2.5. BET Analysis

**Figure 6:**
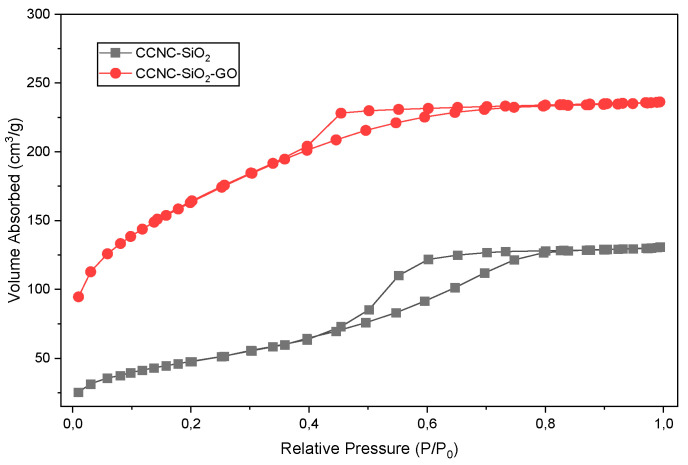
Nitrogen adsorption and desorption isotherms plots of CCNC-SiO_2_ and CCNC-SiO_2_-GO porous monoliths.

Figure 6 illustrates the nitrogen adsorption–desorption isotherms for the CCNC-SiO_2_ and CCNC-SiO_2_-GO porous monoliths. Both isotherms are categorized as Type IV, indicating the presence of a distinct H3-type hysteresis loop. Mesoporous materials typically possess pores ranging from 2 to 50 nm in width and are frequently observed in plate-like particles or aggregates [42]. The hysteresis loop, occurring when the relative pressure (P/P_0_) exceeds 0.4, indicates the condensation of nitrogen within the mesopores. This is characteristic of materials exhibiting a broad spectrum of pore dimensions [43].

Table 2 presents an overview of the textural parameters derived from these isotherms. The incorporation of graphene oxide (GO) into the CCNC–silica matrix significantly enhanced the material’s surface characteristics. The specific surface area significantly increased from 312 m^2^/g for the CCNC-SiO_2_ porous monolith to 512 m^2^/g for the CCNC-SiO_2_-GO hybrid composite. The significant increase is attributed to the extensive theoretical surface area of each GO sheet, which prevents excessive aggregation of silica and cellulose during the sol–gel and drying processes. This results in a more intricate and open porous network [44,45]. The overall pore volume significantly increased from 0.12 cm^3^/g to 0.37 cm^3^/g. The tripling of volume indicates that GO functions as a spacer, hence enlarging the void volumes within the porous monolith structure. The average pore width increased from 1.15 nm, indicating the material was at the threshold of microporous and mesoporous classification, to 4.24 nm. This indicates that the hybrid composite has distinctly transitioned to a mesoporous structure. This high-surface-area, mesoporous structure is advantageous for adsorption applications due to its numerous active sites for pollutant binding and its facilitation of metal ion mobility, such as Cu(II), through the material’s pores [37]. The BET analysis revealed that the incorporation of GO significantly enhanced the textural properties of the porous monolith, increasing the specific surface area from 312 to 512 m^2^/g and the pore volume from 0.12 to 0.37 cm^3^/g. This mesoporous structure (average pore width = 4.24 nm) facilitates the rapid diffusion of hydrated Cu(II) ions (∼0.8 nm) into the adsorbent matrix, thereby improving accessibility to internal active sites and enhancing adsorption kinetics and capacity.

The BET analysis indicated that the incorporation of GO transformed the porous monolith into a mesoporous-dominated structure characterized by a high specific surface area (512 m^2^/g), a substantial pore volume (0.37 cm^3^/g), and an average pore width of 4.24 nm. The textural characteristics are crucial for enhanced Cu(II) absorption. The substantial pore volume provides the material with the physical capacity to retain a significant quantity of Cu(II) ions, hence enhancing its adsorption capability. The extensive surface area ensures a multitude of active sites for metal ion binding. The average pore width of 4.24 nm facilitates the rapid diffusion of hydrated Cu(II) ions ([Cu(H_2_O)_6_]^2+^, ~0.8 nm) into the center of the porous monolith through its wide channels. This diminishes diffusion resistance, accelerates the adsorption process, and ensures that the active sites are not solely located on the object’s exterior.

### 2.6. Factors Affecting Copper Adsorption

#### 2.6.1. Effect of Contact Time

**Figure 7:**
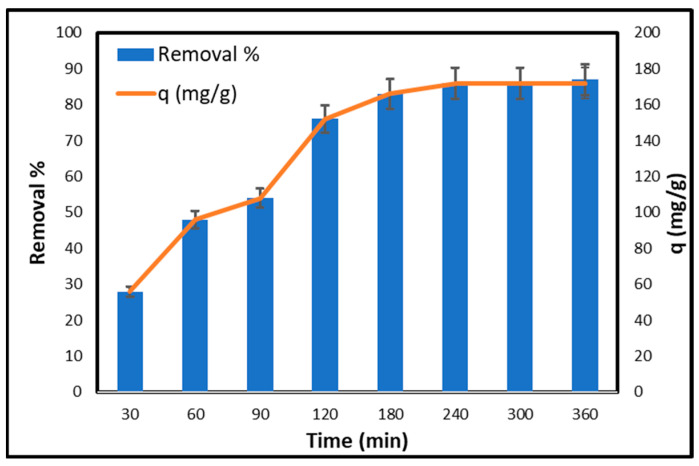
Effect of contact time on the adsorption of copper on CCNC–silica–GO porous monolith [Conc. 200 mg/L, Volume 50 mL, Mass 0.05 g, Temperature 25 °C].

Figure 7 illustrates the effect of contact duration on the removal % and adsorption capacity (q) of copper using CCNC–silica–GO porous monolith. After an initial contact period of 30 min, the removal percentage is 28%, with an adsorption capacity of 56 mg/g, indicating that the adsorption process has begun, although many active sites remain vacant. Extending the contact time to 60 min markedly improves the removal percentage and adsorption capacity, achieving values of 48% and 96 mg/g, respectively, due to the increased adsorption of copper ions. At 90 min, the removal percentage rises to 54%, and the adsorption capacity attains 108 mg/g. The gradual rate of increase suggests that the adsorption sites are becoming increasingly occupied. A contact time of 120 min results in a notable increase in removal percentage (76%) and adsorption capacity (152 mg/g), indicating significant adsorption activity. The rapid initial adsorption observed within the first 120 min can be attributed to the high surface area and mesoporous nature of the CCNC-SiO_2_-GO monolith, as confirmed by BET analysis. The wide pore channels (4.24 nm) allow for efficient intraparticle diffusion of Cu(II) ions, reducing mass transfer resistance and enabling faster uptake. This structural advantage aligns with the high experimental adsorption capacity of 172 mg/g. At 180 min, the removal percentage reaches 83%, with an adsorption capacity of 166 mg/g, indicating that the adsorption process is nearing saturation. After 240 min, the removal percentage and adsorption capacity had increased to 86% and 172 mg/g, respectively. The rate of augmentation begins to stabilize. At 300 min, both levels remain constant at 86% and 172 mg/g, indicating that equilibrium has been attained. After 360 min, the removal percentage increases marginally to 87%, while the adsorption capacity remains constant at 172 mg/g. This indicates that the adsorption process is complete and the porous monolith has attained its maximal adsorption capacity. Initially, extended contact durations result in significant enhancements in both the removal % and the adsorption capacity. When contact durations exceed 240 min, the adsorption process approaches equilibrium, indicating that all active sites of the porous monolith are saturated.

#### 2.6.2. Effect of pH

**Figure 8:**
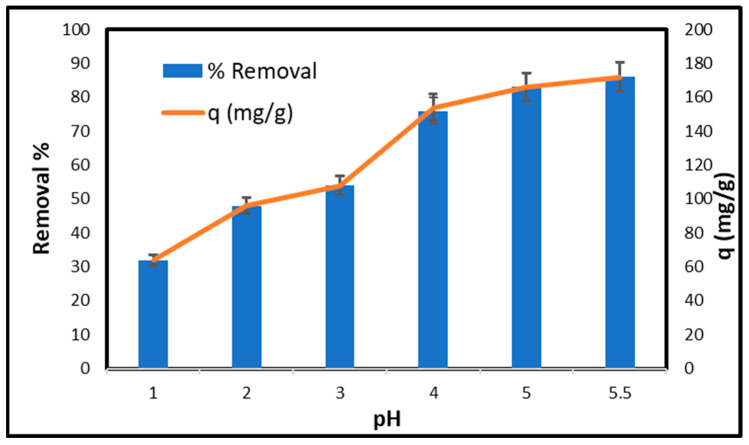
Effect of pH on the adsorption of copper on CCNC–silica–GO porous monolith [Conc. 200 mg/L, Volume 50 mL, time 240 min, Mass 0.05 g, and Temperature 25 °C].

Figure 8 illustrates the relationship between pH and the capacity for copper removal, as well as the retention capacity of CCNC–silica–GO porous monolith. The removal efficiency is 32% at pH 1, while the adsorption capacity is 64 mg/g. This occurs due to the abundance of hydrogen ions (H^+^) in the solution, which compete with copper ions for adhesion sites. As the pH increases to 2, the removal percentage escalates to 48%, while the adsorption capacity reaches 96 mg/g. The reduced concentration of hydrogen ions decreases the likelihood of competition among copper ions, facilitating their adhesion to the surface. At pH 3, the removal efficiency rises to 54%, and the adsorption capacity reaches 108 mg/g. The removal percentage (76%) and adsorption capacity (152 mg/g) significantly increase at pH 4. The reduced acidity facilitates the adhesion of copper ions to surfaces. The removal percentage increases to 83% at a pH of 5, while the adsorption capacity reaches 166 mg/g. The removal percentage increases to 86% at pH 5.5, with a maximum adsorption capacity of 172 mg/g. The provided pH level is optimal for the adhesion of copper ions to surfaces, thereby inhibiting the attachment of hydrogen ions. Raising the pH from 1 to 5.5 markedly improves copper removal and enhances its adsorption capacity. Optimal efficiency is achieved at pH 5.5, yielding a removal rate of 86% and an adsorption capacity of 172 mg/g.

#### 2.6.3. Effect of Initial Copper Concentration

**Figure 9:**
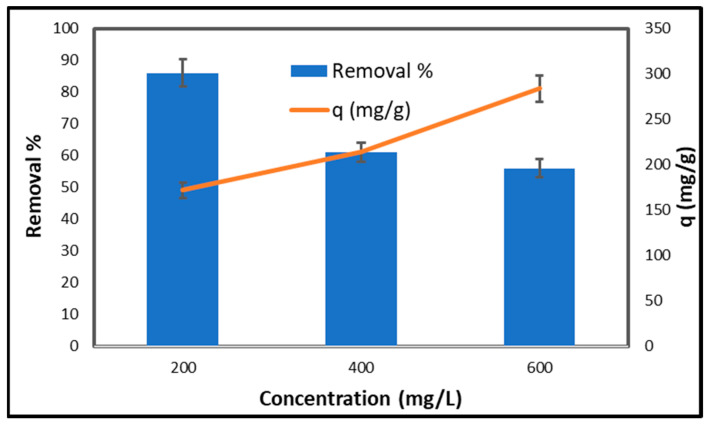
Effect of initial concentration of copper on the adsorption of CCNC–silica–GO porous monolith [Volume 50 mL, pH 5.5, Mass 0.1 g, time 240 min and Temperature 25 °C].

Figure 9 illustrates the impact of the initial copper concentration on both the percentage of copper removed and the adsorption capacity of CCNC–silica–GO porous monolith for copper. At an initial concentration of 200 mg/L, the clearance percentage is 86%, and the adsorption capacity is 172 mg/g. Porous monolith demonstrates high efficiency owing to its numerous active sites, facilitating significant absorption of copper ions at low concentrations. However, as the initial concentration increases to 400 mg/L, the removal percentage decreases to 61%, and the adsorption capacity decreases to 214 mg/g. This reduction indicates that the availability of active sites becomes a limiting factor as concentration increases, hence decreasing adsorption efficiency [46]. At an initial concentration of 600 mg/L, the removal percentage further declines to 56%, while the adsorption capacity is 284 mg/g. This indicates that the adsorption sites are nearly saturated. Despite the increased concentration, the porous monolith’s capacity to absorb copper ions decreases, rendering it less efficient. Generally, as the initial concentration of copper increases, the proportion of copper removed decreases due to the saturation of adsorption sites. The adsorption capacity decreases, indicating that the porous monolith can only absorb copper ions at reduced concentrations [47].

#### 2.6.4. Effect of Adsorbent Dose

**Figure 10:**
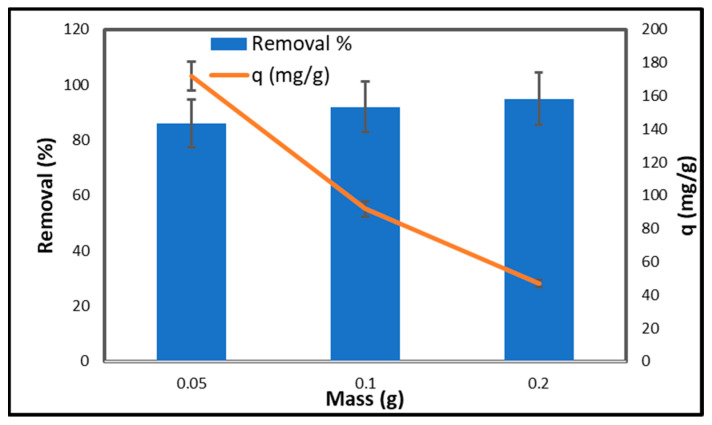
Effect of adsorbent dose on the adsorption of copper on CCNC–silica–GO porous monolith [Conc. 200 mg/L, Volume 50 mL, time 240 min, pH 5.5, and Temperature 25 °C].

Figure 10 illustrates the correlation between the quantity of adsorbent and the percentage of copper removal, along with the adsorption capacity (q) of CCNC–silica–GO porous monolith. An adsorbent dose of 0.05 g achieves a removal efficiency of 86%, indicating that the adsorbent capacity is 172 mg/g. This indicates that copper can be effectively adsorbed with a minimal quantity of adsorbent. Increasing the adsorbent dose to 0.1 g results in a removal percentage of 92%, while the adsorption capacity decreases to 92 mg/g. This indicates that while the total quantity of copper extracted increases, the copper adsorbed per gram of porous monolith decreases. This is likely due to an increased number of surfaces for copper adhesion, resulting in a higher removal percentage but a diminished concentration of copper per unit mass. At an adsorbent dosage of 0.2 g, the removal percentage increases to 95%, while the adsorption capacity decreases to 47 mg/g. The data indicates that removal efficiency improves with increasing adsorbent dosage. This is due to the increased availability of active sites for copper ions to adhere to [48]. The adsorption capacity per unit decreases as the copper ions are distributed across a greater number of adsorption sites. Higher doses of adsorbent enhance the removal percentage; however, they also induce a dilution effect that reduces the adsorption capacity per gram of the adsorbent. Determining the optimal quantity of adsorbent is crucial for achieving maximum removal efficiency while maintaining sufficient adsorption capacity.

#### 2.6.5. Effect of Temperature

**Figure 11:**
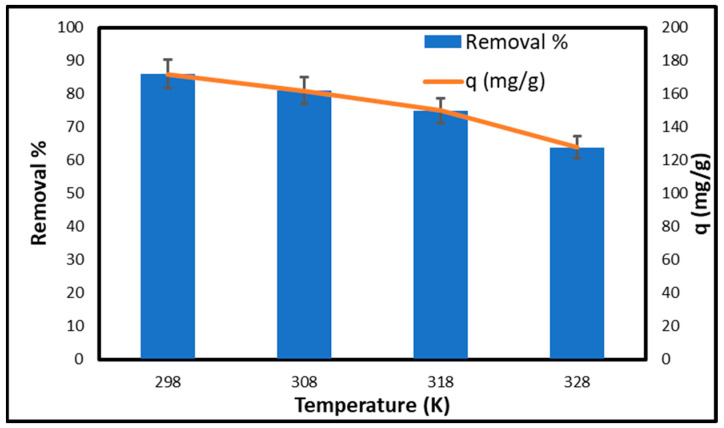
Effect of temperature on the adsorption of copper on CCNC–silica–GO porous monolith [Conc. 200 mg/L, Volume 50 mL, pH 5.5, and Mass 0.05 g.].

Figure 11 illustrates the influence of temperature on the removal percentage and adsorption capacity (q) of copper utilizing CCNC–silica–GO porous monolith. At 298 K, the removal percentage is high at 86%, with an adsorption capacity of 172 mg/g, indicating that lower temperatures favor the adsorption process. At a temperature of 308 K, the removal percentage decreases to 81%, and the adsorption capacity reduces to 162 mg/g. The trend persists at 318 K, with the removal rate decreasing to 75% and the adsorption capacity declining to 150 mg/g. The removal percentage decreases to 64% at 328 K, while the adsorption capacity is 128 mg/g. The results demonstrate that increased temperatures negatively affect the adsorption efficiency of copper onto the CCNC–silica–GO porous monolith. The reduction in removal percentage and adsorption capacity with rising temperature suggests that the adsorption process is exothermic. As the temperature rises, the kinetic energy of the copper ions increases, reducing their interaction with the adsorbent sites on the porous monolith and leading to lower adsorption efficiency. The data indicates that the adsorption of copper using CCNC–silica–GO porous monolith is more effective at lower temperatures and less effective at higher temperatures.

### 2.7. Adsorption Isotherm

Adsorption isotherm models were utilized to assess the adsorption efficiency of CCNC–silica–GO porous monolith and to investigate the interactions between the adsorbent and the adsorbate [47]. The Langmuir and the Freundlich models are among the most frequently utilized isotherm models in the literature [48,49]. The Langmuir model posits that adsorption takes place on a uniform surface with monolayer coverage, as demonstrated by the following equation:

(1) $$\frac{C_{e}}{q_{e}}=\frac{C_{e}}{q_{m}}\;+\;\frac{1}{q_{m}k_{L}}$$

In this context, *Ce* (mg/L) refers to the concentration of copper at equilibrium, while *qe* (mg/g) denotes the quantity of copper adsorbed onto the surface of CCNC–silica–GO porous monolith at equilibrium. The parameter *q*_*m*_ (mg/g) refers to the maximum adsorption capacity of copper on the adsorbent surface, and *K*_*L*_ (L/mg) is the Langmuir constant. The values of *q*_*m*_ and *K*_*L*_ were obtained from the slope and intercept of the plot of Ce/*q*_*e*_ versus *C*_*e*_. Furthermore, the equilibrium parameter *R*_*L*_ was calculated using the following equation to determine if the adsorption process is favorable [47].

(2) $$R_{L}=\frac{1}{\left(1+K_{L}C_{0}\right)}$$

In this scenario, *C*_0_ represents the initial quantity of copper. The Freundlich model, indicating that the adsorbent surface is heterogeneous, can be expressed as:

(3) $$\ln qe=\ln K_{F}+\frac{1}{n}\ln C_{e}$$

**Figure 12:**
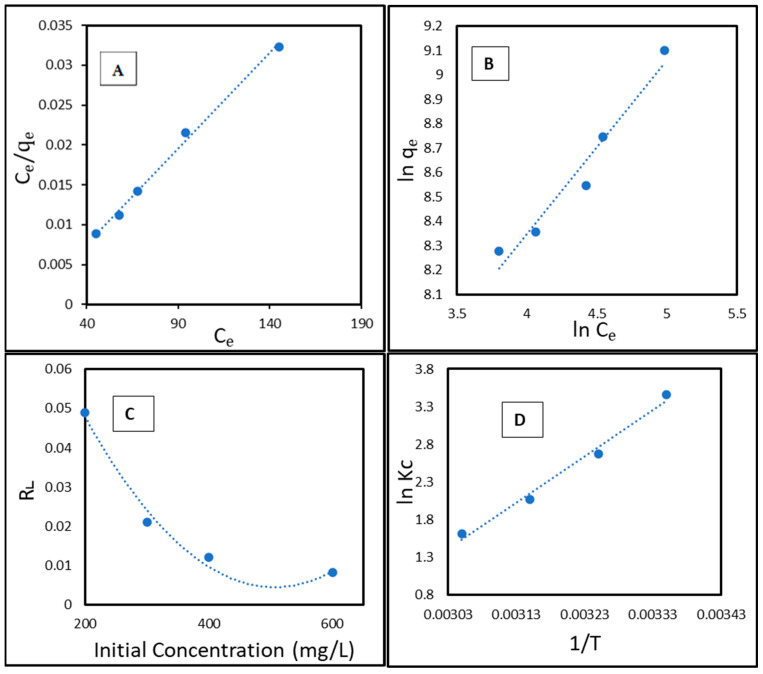
(**A**) Langmuir isotherm model, (**B**) Freundlich isotherm model, (**C**) Separation factor, and (**D**) van’t Hoff plot for the adsorption of copper onto CCNC–silica–GO porous monolith.

The Freundlich constants, *KF* (adsorption capacity) [mg^1−1/n^ L^1/n^ g^−1^] and *n* (adsorption intensity), are obtained from the intercept and slope of the ln *q*_*e*_ versus ln *C*_*e*_ plot. Figure 12A,B presents the fitting plots for the Langmuir and the Freundlich adsorption isotherms, respectively. Table 3 provides an overview of the parameters generated by these models. The Langmuir isotherm exhibited a superior determination coefficient (R^2^) compared to the Freundlich isotherm (Table 4). The adsorption of copper onto CCNC–silica–GO porous monolith aligns more closely with the Langmuir model. The theoretical *qm* derived from the Langmuir model closely aligns with the experimentally measured value. Figure 12C illustrates the *R*_*L*_ values obtained from Equation (6) plotted against the initial copper concentration. The *R*_*L*_ values ranged from 0.0091 to 0.052, indicating an interaction between copper molecules and the adsorbent. The *R*_*L*_ values, approaching zero, indicate that copper can be readily adsorbed onto CCNC–silica–GO porous monolith across a broad spectrum of initial copper concentrations.

### 2.8. Thermodynamic Studies

The impact of temperature fluctuations on the adsorption of copper onto CCNC–silica–GO porous monolith was evaluated, and the characteristics of the adsorption process were investigated. The subsequent equations were employed to determine the key thermodynamic parameters (ΔH°, ΔS°, ΔG°) [47].

(4) $$lnKc=\left(\frac{∆S{}^{\circ}}{R}\right)-\left(\frac{∆H{}^{\circ}}{RT}\right)$$

(5) ∆G°=−RT lnKc

Kc represents the equilibrium constant and is dimensionless. ΔH°, ΔG°, and ΔS° denote the standard changes in enthalpy, Gibbs free energy, and entropy, respectively [49]. The universal gas constant, R, is valued at 8.314 × 10^−3^ KJ·mol^−1^·K^−1^. T represents the temperature measured in Kelvin (K). The slope and interception of the ln Kc versus 1/T plot (Figure 12D) were utilized to determine the values of ΔH° and ΔS°. Table 5 presents the thermodynamic values associated with the adhesion of copper to CCNC–silica–GO porous monolith. The decrease in Kc values with rising temperature, coupled with a negative ΔH°, indicates that the process is exothermic. The magnitude of ΔH° varies according to the forces involved in the adsorption process. ΔH° values ranging from 2 to 40 kJ/mol indicate the presence of van der Waals forces, dipole interactions, hydrogen bonding, and/or coordination exchange. Values exceeding 60 kJ/mol indicate the formation of chemical bonds. The ΔH° value of −37.24 kJ/mol signifies that the adsorption process is exothermic. This value typically falls within a range indicative of physical processes, such as electrostatic interactions. However, it may also signify insufficient chemical coordination. The pseudo-second-order kinetics and FTIR evidence of carboxylate group participation unequivocally indicate that specialized coordination to functional groups is an essential event. The total adsorption enthalpy probably indicates a blend of strong, specific coordination bonds with carboxylate groups and weaker, non-specific electrostatic interactions across the porous monolith’s wide surface. The decrease in entropy (ΔS°, −105.25 J/mol·K) indicates that the process is more favorable at lower temperatures. The observation that the negative values of ΔG° decrease with increasing temperature indicates that the process exhibits greater spontaneity at lower temperatures.

### 2.9. Kinetic Studies

To gain a deeper insight into the adsorption process of copper onto CCNC–silica–GO porous monolith, the widely used pseudo-first-order, pseudo-second-order, and intra-particle diffusion kinetic models were applied and are described as follows [47,48]:

(6) $$\ln\left(q_{e}-q_{t}\right)=\ln q_{e}-\;k_{1}\;.t$$

(7) $$\frac{t}{q_{t}}=\frac{1}{K_{2}}+\frac{t}{q_{e}}$$

*q*_*e*_ represents the equilibrium adsorption amount, while *q*_*t*_ (mg/g) denotes the amount of copper adsorbed at time *t*. The first-order rate constant is indicated by *k*_1_ (min^−1^), and the second-order rate constant is represented by *k*_2_ (g/mg·min). The intra-particle diffusion rate constant is denoted as *k*_*id*_ (mol/g·min^1/2^), and *C* signifies the boundary layer thickness.

**Figure 13:**
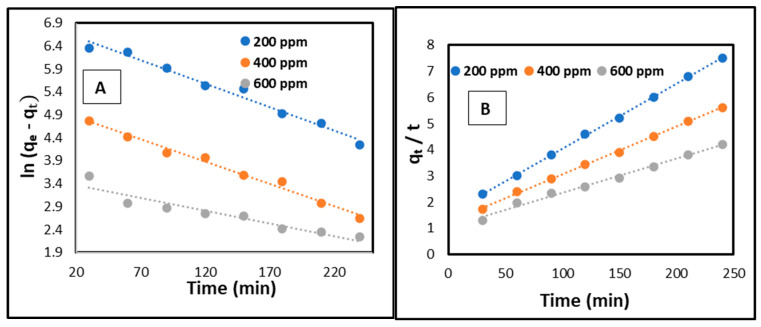
(**A**) Kinetic models showing pseudo-first order, (**B**) Kinetic models showing pseudo-second order for the adsorption of copper onto CCNC–silica–GO porous monolith.

Figure 13A,B present the linear plots corresponding to the pseudo-first order and pseudo-second order models, respectively. Table 6 presents a summary of the parameters identified in these plots. The analysis of determination coefficients (R^2^) revealed that the pseudo-first-order model (R^2^ = 0.921–0.941) exhibited inferior accuracy relative to the pseudo-second-order model (R^2^ = 0.992–0.995). The equilibrium capacity values (*q*_*e*_, *cal*) derived from the pseudo-second-order model exhibited greater proximity to the experimental values (*q*_*e*_, *exp*) compared to those obtained from the pseudo-first-order model. The pseudo-second-order model more accurately describes the adsorption of copper onto CCNC–silica–GO porous monolith.

### 2.10. Elucidation of the Cu(II) Sorption Mechanism

The exceptional capacity of the CCNC-SiO_2_-GO porous monolith to adsorb Cu(II) ions results from a synergistic multi-mechanism process facilitated by certain functional groups on its surface. The carboxylate groups (-COO^−^) on the carboxylated cellulose nanocrystals serve as the primary active binding sites [50]. They effectively coordinate with Cu(II) to create stable inner-sphere complexes, as evidenced by FTIR studies, the Langmuir isotherm, and pseudo-second-order kinetics [51]. Supplementary interactions involve hydroxyl groups from the CCNCs, silica matrix, and graphene oxide. These groups collaborate via diminished coordination and electrostatic attraction, particularly at the optimal pH of 5.5, when the surface exhibits a negative charge. The process commences with ions rapidly diffusing into the mesoporous porous monolith framework. Subsequently, they are electrostatically pre-concentrated on the negatively charged surface and ultimately chemisorbed via complexation with carboxylate groups. GO enhances performance further by offering additional coordination locations and reinforcing the framework. The amalgamation of CCNC, SiO_2_, and GO constitutes a highly efficient and adaptable system for the removal of Cu(II).

### 2.11. Cellulose-Based Silica Composite Porous Monolith Reusability

**Figure 14:**
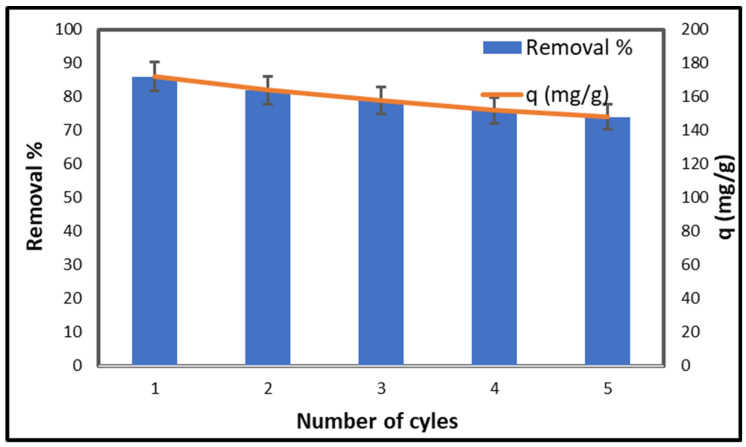
Reusability of CCNC silica GO composite porous monolith for the adsorption of copper [Conc. 200 mg/L, Volume 50 mL, pH 5.5, Mass 0.05 g, and Temperature 25 °C].

The stability and regenerability of an adsorbent are essential for economic viability, rendering reusability a critical factor. Considering this, CCNC–silica–GO porous monolith underwent multiple cycles of adsorption and desorption testing. Figure 14 indicates that the removal efficiency remained above 70%, while the adsorption capacity peaked at 148 mg/g following five consecutive cycles. This demonstrates the stability of the synthesized CCNC–silica–GO porous monolith and its potential for reuse.

### 2.12. Comparison of Adsorption Capacity and Reusability with Other Reported Materials

To contextualize the performance of the CCNC-SiO_2_-GO porous monolith, its maximum Cu(II) adsorption capacity (172 mg/g) and reusability (>70% after 5 cycles) were compared with those of other recently developed adsorbents, including those based on cellulose, silica, and graphene oxide (Table 7). While direct comparisons must be made cautiously due to differing experimental conditions, the synthesized porous monolith demonstrates a highly competitive and superior adsorption capacity. Its performance surpasses that of many pure or binary composite materials and is comparable to some of the most effective ternary composites reported. More importantly, the combination of a high adsorption capacity derived from sustainable sugarcane bagasse with excellent reusability in a monolithic, easy-to-handle porous monolith form highlights a key advantage of the proposed CCNC-SiO_2_-GO hybrid system. This combination of high performance, structural integrity, and regenerability positions it as a highly promising candidate for practical wastewater treatment applications.

## 3. Conclusions

A hybrid composite porous monolith (CCNC-SiO_2_-GO) was synthesized, characterized, and applied for the efficient adsorption of copper (Cu(II)) ions from aqueous solutions. Sugarcane bagasse, an economical and sustainable agricultural byproduct, was employed for cellulose extraction and the synthesis of CCNCs to improve metal reactivity and adhesion. Silica was combined with graphene oxide via a sol–gel process to create a composite porous monolith that demonstrates improved structural and adsorptive characteristics.

The synthesis of the hybrid composite was confirmed through FTIR, XRD, SEM, TGA, and BET analyses. Adsorption necessitates the presence of functional groups such as -COOH, Si-O-Si, and C-O-C, as evidenced by FTIR spectra. XRD measurements indicated a reduction in crystallinity with each modification step, resulting in an amorphous composite that facilitates the identification of adsorption sites. SEM revealed the transformation of bagasse from a coarse, fibrous structure to a porous, three-dimensional network within the porous monolith. The TGA results indicated that the composite exhibited thermal stability, thus rendering it appropriate for environmental applications. A BET study demonstrated that the incorporation of GO enhanced the specific surface area from 312 m^2^/g to 512 m^2^/g and augmented pore capacity. This demonstrated that a mesoporous structure is capable of capturing pollutants.

The CCNC-SiO_2_-GO porous monolith demonstrated remarkable adsorption capabilities. Optimal conditions (pH 5.5, 25 °C) facilitate the retention of Cu(II) ions at a rate of 172 mg/g. Adsorption exhibited sensitivity to initial concentration, contact duration, pH level, adsorbent quantity, and temperature. The adsorption mechanism was most accurately characterized by pseudo-second-order kinetics, signifying that the rate-limiting phase was governed by chemisorption, particularly the coordination of Cu(II) ions to the carboxylate groups on the CCNCs. FTIR research backed this up much more. The thermodynamic characteristics indicate that this chemisorption is significantly influenced by exothermic physical adsorption (e.g., electrostatic attraction), yielding an overall enthalpy value of −37.24 kJ/mol. So, the porous monolith’s high efficiency comes from a synergistic process that combines strong, specific coordination with carboxylate ligands and fast, non-specific electrostatic attraction made possible by the material’s high surface area and porosity. The Langmuir model elucidates equilibrium isotherm data, indicating that a monolayer adsorbs onto a planar surface. The thermodynamic parameters (ΔG° < 0, ΔH° < 0, ΔS° < 0) indicate that adsorption at the solid-solution interface is spontaneous, exothermic, and associated with a decrease in entropy. The porous monolith demonstrated significant reusability, maintaining over 70% of its removal efficacy after five cycles.

A notable constraint of this study is that the adsorption efficacy was evaluated exclusively using single-component Cu(II) solutions. In actual wastewater, many competing ions, including Zn(II), Pb(II), Ni(II), and Ca(II), are commonly present, which may influence the selectivity and effectiveness of the adsorbent by competing for the available active sites. The Langmuir model suggests a significant affinity and the presence of carboxylate groups, indicating potential for selective metal binding; however, further work is necessary. Consequently, additional research is necessary to thoroughly assess the selectivity of the CCNC-SiO_2_-GO porous monolith in multi-metal systems. This will entail performing competitive adsorption tests and calculating distribution coefficients (Kd) to evaluate its affinity for Cu(II) relative to other prevalent cations. These experiments are essential for precisely evaluating the material’s capacity for targeted metal recovery and its efficacy in intricate, real-world water matrices.

This study indicated that the CCNC-SiO_2_-GO hybrid porous monolith effectively removes toxic copper ions from wastewater, showing both durability and reusability. The combination of CCNCs, silica, and GO shows promise for use in environmental remediation and water treatment applications. 

## 4. Materials and Methods

### 4.1. Materials

Sugarcane bagasse (SCB) waste was supplied by the sugar company at Empangeni (Felixton) in KwaZulu Natal, South Africa. Hydrochloric Acid (32%), Graphite, Hydrogen peroxide (30%), Sulfuric acid (98%), Sodium Nitrate, Sodium Hydroxide pellets, were purchased from Laboratory Supplies and used as received. Acetic acid, Copper(II) nitrate trihydrate (Cu(NO_3_)_2_·3H_2_O), Sodium chlorite 80%, Ammonium persulphate, Ammonia 25%, Tertiary butyl alcohol, Potassium permanganate, and Tetraethyl orthosilicate 98% were purchased from Prestige Laboratories South Africa.

### 4.2. Extraction of Cellulose

The sugarcane bagasse (SCB) was mechanically processed with a Fritsch cutting mill pulverizer 15. Thereafter, it was boiled in distilled water for two hours and subsequently dried in an oven at 55 °C overnight. The desiccated SCB was subjected to a chemical treatment with 4 wt% sodium hydroxide (NaOH) at 80 °C for 1 h, subsequently rinsed with distilled water, and the procedure was repeated twice. The alkali-treated SCB was subsequently dried in an oven at 55 °C.

The alkali-treated SCB underwent a bleaching process utilizing a buffer solution consisting of 54 g of NaOH, 150 mL of acetic acid (CH_3_COOH), and 2 L of 1.7 wt% sodium chlorite (NaClO_2_) solution for 1 h at 80 °C, a procedure that was repeated twice. The bleached cellulose was subsequently filtered, rinsed with distilled water until a neutral pH was attained, and dried overnight at 55 °C. The yield of the extracted cellulose was calculated based on the dry mass of the starting SCB material and was found to be approximately 31%.

### 4.3. Preparation of Carboxylate Cellulose Nanocrystals

An 18 g dry mass of cellulose was added to a 600 mL solution of 2 M ammonium persulfate (APS). The mixture underwent ultrasonication at high frequency with continuous stirring for four hours at 70 °C. Following the attainment of a homogeneous dispersion, the mixture was subjected to centrifugation at 6000 rpm with distilled water, a procedure that was repeated four times. Thereafter, the suspension was dialyzed with distilled water until the carboxylate cellulose nanocrystal (CCNC) solution reached a near-neutral pH.

### 4.4. Preparation of CCNC–Silica Hydrogel

A mixture consisting of 2.8 mL of 1.7 wt% carboxylate cellulose nanocrystal (CCNC) dispersion (equivalent to 0.12 g dry cellulose), 9.2 mL of water, 2 mL of tetraethyl orthosilicate (TEOS) (98% purity, 1.9 g, 9.1 mmol), and 0.16 mL of aqueous HCl (0.29 M, 46 µmol HCl) was stirred overnight to induce the hydrolysis of TEOS, resulting in a CCNC–silica sol. Subsequently, 0.85 mL of 0.1 mol/L NH_3_ (85 µmol) was rapidly added and thoroughly mixed to initiate the condensation process. The solution was then transferred into syringes to form cylindrical hydrogels, with the option to adjust shapes using different molds. After complete gelation, the silica nanocomposites were aged in water at 50 °C for at least 10 h to enhance the stiffness of the silica gel network. The hydrogels were kept in distilled water in a fridge until further use.

### 4.5. Preparation of Graphene Oxide

Graphene oxide was synthesized using a modified Hummer’s method [52]. Approximately 1 g of graphite was dispersed in 80 mL of 98% sulfuric acid under continuous stirring. Next, 0.5 g of sodium nitrate (NaNO_3_) was added at 4 °C and stirred for 1 h, followed by the gradual addition of 5 g of potassium permanganate (KMnO_4_). The mixture was stirred for an additional hour, then the temperature was increased to 45 °C and maintained for 40 min. Subsequently, 100 mL of distilled water was added, and the temperature was raised to 90–100 °C and maintained for 2 h. To terminate the reaction, 300 mL of distilled water and 20 mL of 30% hydrogen peroxide were added. The resulting black product was filtered, washed with 10% hydrochloric acid (HCl) and distilled water, and then dried in an oven at 50 °C overnight.

### 4.6. Preparation of CCNC–Silica–GO Hybrid Composite Porous Monolith

The previously prepared CCNC–silica hydrogel (refer to Section 4.4) was immersed in a 2 wt% graphene oxide (GO) sol–gel solution (refer to Section 4.5) at room temperature for 24 h while being stirred on a laboratory shaker. The mixture was then subjected to sonication at 70 °C for 4 h to achieve adsorption equilibrium. The synthesized CCNC–silica–GO hybrid composite hydrogel was thoroughly rinsed with distilled water and tertiary butyl alcohol (TBA) to remove unreacted residues. To minimize capillary stress during drying, the water within the hydrogel was exchanged with tertiary butyl alcohol (TBA) by soaking the hydrogel in TBA for 24 h, with the TBA being replaced three times. Finally, the CCNC–silica–GO hybrid composite porous monolith was obtained by vacuum drying at 30 °C for period of 24 h. The synthesis yield for the final CCNC-SiO_2_-GO hybrid composite porous monolith was determined based on the mass of the dry solid product relative to the total mass of the precursors used (dry CCNC, TEOS, and GO). The average yield for the porous monolith synthesis was 79%, indicating a highly efficient sol–gel and integration process with minimal mass loss.

While this material was not produced via supercritical drying, the characterization data confirms that the solvent exchange with TBA and gentle vacuum drying successfully preserved a highly porous network. The resulting monolith exhibited the key aerogel-like properties of a high specific surface area (512 m^2^/g, see Section 2.5) and a low-density, interconnected mesoporous structure (as seen in SEM, Section 2.3), justifying its classification as a high-performance porous monolith suitable for adsorption applications.

### 4.7. Characterization Methods

#### 4.7.1. Fourier-Transform Infra-Red Spectroscopy

FTIR analysis was performed using a Perkin Elmer (Waltham, MA, USA) attenuated total reflection (ATR) FTIR spectrometer (Perkin Elmer UATR Two) equipped with a diamond crystal. For sample preparation, a small amount of each solid sample (raw SCB, extracted cellulose, CCNC, CCNC-SiO_2_ porous monolith, GO, and CCNC-SiO_2_-GO porous monolith) was placed directly onto the diamond ATR crystal. A consistent pressure was applied using the instrument’s built-in anvil to ensure good contact between the sample and the crystal for all measurements. Spectra were acquired in the range of 4000 to 500 cm^−1^ with a resolution of 4 cm^−1^, and each spectrum represents the average of 32 scans to ensure a high signal-to-noise ratio. A background spectrum was collected with a clean crystal before each sample measurement. All spectra were baseline-corrected and normalized within the instrument’s software to enable a direct qualitative comparison of the functional groups present in the different materials.

#### 4.7.2. X-Ray Diffraction

X-ray diffraction (XRD) analysis of the materials was conducted utilizing a Bruker AXS Advance D8 diffractometer situated in Karlsruhe, Germany. The apparatus, functioning at 40 kV and 40 mA at ambient temperature, was fitted with a monochromatic Cu Kα X-ray source (λ = 1.5406 Å). The crystallinity index (CI) was assessed utilizing the Segal empirical approach and the deconvolution method. The Segal empirical technique computes the crystallinity index (CI) using the heights of I_002_ and I_min_, identified between the 002 and 001 peaks. The CI computation according to the methodology outlined below [53]:

(8) $$CI\%=\frac{I_{002}-I_{am}}{I_{002}}\times 100$$

In this context, I_002_ represents the peak’s maximum diffraction intensity, while I_am_ denotes the diffraction intensity of the amorphous material [53].

The deconvolution method determines the CI by calculating the ratio of the combined area of all crystalline peaks to the total area under the diffraction curve.

(9) $$CI\%=\frac{ƩAcryst}{ƩAcryst+ƩAamorp}\times 100$$

In this context, A_cryst_ represents the area corresponding to the crystalline domain, whereas A_amorp_ denotes the area associated with the amorphous domain.

#### 4.7.3. Scanning Electron Microscopy

SEM analysis of the samples was performed using an FEI Quanta 200 electron microscope (FEI, Hillsboro, OR, USA) operating at an acceleration voltage of 20 kV. Before analysis, the samples were carbon-coated using Edward’s E306A coating system (Edwards, London, UK).

#### 4.7.4. Thermogravimetric Analysis

Samples were subjected to thermogravimetric analysis (TGA) using a Perkin Elmer Pyris 6 TGA analyser. Samples weighing between 10 and 15 mg were heated from 35 to 700 °C at a rate of 5 °C per minute. The analysis was conducted in a nitrogen atmosphere with a flow rate of 20 mL.min^−1^.

### 4.8. Adsorption Experiments

Batch adsorption tests were conducted to evaluate the efficacy of the adsorption procedure. We added precise quantities of porous monoliths (0.05 to 0.2 g) to 50 mL of copper solutions (derived from Cu(NO_3_)_2_·3H_2_O) with concentrations ranging from 200 to 600 mg/L in 100 mL Erlenmeyer flasks. We employed 0.1M HCl or NaOH solutions to adjust the pH from 2 to 6. The flasks were thereafter placed in an incubator shaker and agitated at a constant speed of 150 rpm at a temperature of 301 K. Samples were extracted from the shaker at intervals of 30, 60, 90, 120, 150, 180, 210, and 240 min. The samples were filtered using a 0.45 µm syringe filter to ensure thorough separation of the monolith from the liquid phase. The concentration of copper remaining in the filtrate was measured at 324 nm using a Varian Spectra AA atomic absorption spectrophotometer. Utilizing Equations (3) and (4), we calculated the removal percentage (R%) and the adsorption capacity (qt).

(10) $$CI\%=\frac{C_{0}-C_{t}}{C_{0}}\times 100$$

(11) $$CI\%=\frac{C_{0}-C_{t}}{M}\times V$$

Here, C_0_ and C_t_ (mg/L) denote the copper concentration at the initial time and at a given time, respectively. V represents the volume of the copper solution (L), and M is the net weight of the dried CCNC-SiO_2_-GO (g). To renew and reuse the copper porous monolith, they were collected and cleaned using a re-generation solution, which is a diluted acid that desorbs copper ions. The porous monolith was then dried at 50 °C in a vacuum oven and used again in the following cycles to evaluate their effectiveness after multiple uses.

## Figures and Tables

**Table 1:** Crystalline Index values of materials studied using Segal and deconvolution methods.

Fiber Type	CI (%) (Segal)	CI (%) (Deconvolution)
Raw SCB	29	27
Extracted cellulose	66	63
CCNC	58	54
CCNC–silica porous monolith	-	42
CCNC–silica–GO porous monolith	-	21

**Table 2:** Physical parameters of the prepared porous monoliths.

Sample	Specific Surface Area (m^2^/g)	Pore Volume (cm^3^/g)	Pore Diameter (nm)
CCNC-SiO_2_	312	0.12	1.15
CCNC-SiO_2_-GO	512	0.37	4.24

**Table 3:** Adsorption study results for cellulose-based silica porous monoliths studied.

Cellulose-Based Silica Porous Monoliths	Removal (%)	Adsorption Capacity (mg/g)
CCNC silica	22	32
CCNC–silica–GO	86	172

**Table 4:** Adsorption isotherm parameters for the adsorption of copper onto CCNC–silica–GO porous monolith.

	Langmuir Model			Freundlich Model		
Material	q_max_ (mg/g)	K_L_ (L/mg)	R^2^	K_F_ (mg^1−1^/ⁿ L^1^/ⁿ g^−1^)	n	R^2^
CCNC–silica–GO	172	0.27	0.993	57.32	3.57	0.908

**Table 5:** Thermodynamic parameters for the adsorption of copper onto CCNC–silica–GO porous monolith.

T (K)	ΔG° (KJ·mol^−1^)	ΔH° (KJ·mol^−1^)	ΔS° (J·mol^−1^·K^−1^)
298	−6.22	−37.24	−105.25
308	−5.32		
318	−4.46		
328	−3.65		

**Table 6:** Kinetic parameters for the adsorption of copper onto CCNC–silica–GO porous monolith.

C_o_ (mg/L)	*q*_*e*, exp_ (mg/g)	Pseudo-First Order			Pseudo-Second Order		
		*q*_*e*, cal_ (mg/g)	*k*_1_ (min^−1^)	R^2^	*q*_*e*, cal_ (mg/g)	*k*_2_ (g/mg min)	R^2^
200	172	155	0.0158	0.941	186.24	0.00028	0.995
400	214	182	0.0154	0.932	130.46	0.00025	0.994
600	284	196	0.0149	0.921	124.62	0.00022	0.992

**Table 7:** Comparison of the maximum Cu(II) adsorption capacity and reusability of the CCNC-SiO_2_-GO porous monolith with other reported adsorbents.

Adsorbent Material	Maximum Capacity (mg/g)	Reusability (Cycles/% Retention)	Key Features/Limitations	Reference
CCNC-SiO_2_-GO Porous monolith (This work)	172	5/>70%	Monolithic porous monolith, high capacity, excellent reusability, from waste biomass.	-
Carboxylated CNC	149.3	4/~85%	High capacity from functionalization, but powdered form.	[12]
Mesoporous Silica (SBA-15)	41.2	-	High surface area, but low intrinsic capacity for Cu(II).	[16]
Graphene Oxide (GO)	117.5	-	High capacity, but prone to aggregation and difficult recovery.	[45]
Chitosan/GO Composite Beads	88.7	5/~85%	Good reusability, but moderate capacity.	[9]
Cellulose/Silica Composite	105.0	-	Binary composite, moderate capacity.	[23]
Alginate/GO/Cellulose Foam	181.8	5/~90%	High capacity and reusability, similar performance tier.	[50]
EDTA-modified CNC/Silica	158.0	4/~80%	High capacity from chelating agent, requires complex modification.	[51]

## Data Availability

The original contributions presented in this study are included in the article. Further inquiries can be directed at the corresponding author.
